# Tophaceous gout

**DOI:** 10.1002/ccr3.9033

**Published:** 2024-06-11

**Authors:** Durga Neupane, Nimesh Lageju, Lokesh Shekher Jaiswal, Narendra Pandit, Asim Mahat, Saransh Ghimire, Upendra Pokhrel, Sefali Koirala

**Affiliations:** ^1^ B.P. Koirala Institute of Health Sciences Dharan Nepal; ^2^ Department of Surgery (Division of CTVS) B.P. Koirala Institute of Health Sciences Dharan Nepal; ^3^ Department of Surgical Gastroenterology Birat Medical College Teaching Hospital (BMCTH) Biratnagar Nepal; ^4^ Kathmandu Medical College and Teaching Hospital Kathmandu Nepal; ^5^ Patan Academy of Health Sciences Lalitpur Nepal; ^6^ College of Dental Surgery, B.P. Koirala Institute of Health Sciences Dharan Nepal

**Keywords:** allopurinol, gout, hyperuricemia, monosodium urate, tophi

## Abstract

Hyperuricemic patients (≥7.8 mg/dL) can develop polyarticular tophaceous gout from intermittent arthritis if untreated. Acute flares and tophi development can be avoided by lowering blood urate levels with xanthine oxidase inhibitors.

A 65‐year‐old male (BMI: 23 kg/m^2^) with chronic hypertension under amlodipine presented with chief complaints of multiple joint swelling on bilateral hands and feet. The swelling appeared 15 years back, and gradually increased over the years. The patient had frequent pain from the swelling in the past, but instead of seeking medical attention, he took over‐the‐counter painkillers. He has no family history of such swellings but has a 35‐year history of chronic alcohol use, smokeless tobacco use, and cigarette smoking. On examination, multiple large, firm, and immobile swellings were located over the proximal and middle phalanges along with the metacarpophalangeal joints of both hands (Figure 1). Similar swellings were present over the metatarsophalangeal joints and ankle joints of both feet (Figure 2). There was no ulceration over the swelling. On laboratory evaluation, his uric acid level was 9.6 mg/dL (N: 2.5–7.8 mg/dL) with a normal renal function test. Plain radiography of both hands and feet revealed significant osteolysis of the involved joint. Needle aspiration yielded white viscous fluid which showed numerous needle‐shaped birefringent crystals of monosodium urate on polarized light. The patient was prescribed Allopurinol 100 mg/day along with counseling on lifestyle modifications. He is on constant follow‐up with us.

**FIGURE 1:**
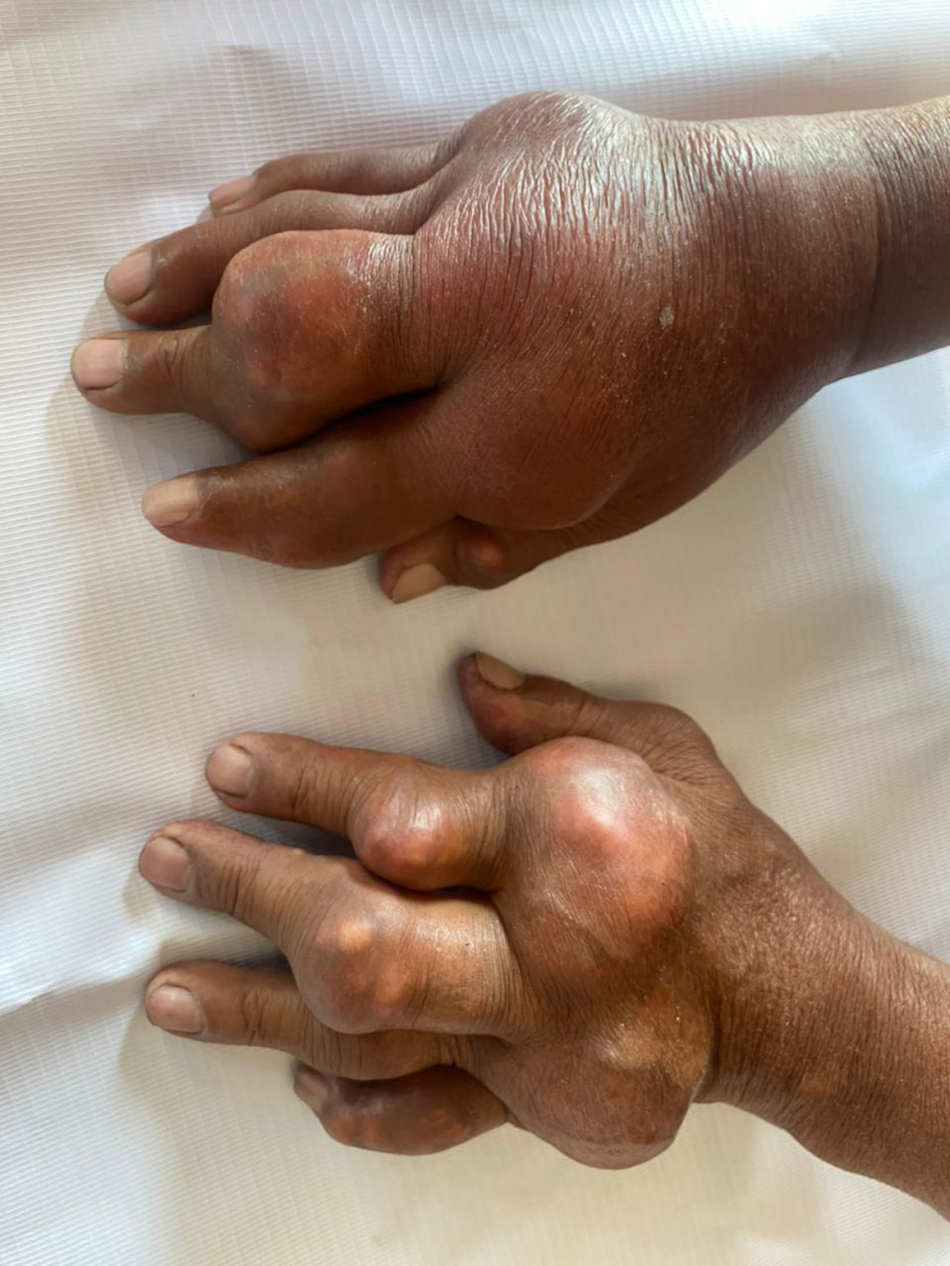
Multiple swellings of the proximal and middle phalanges along with metacarpophalangeal joints of both hands.

**FIGURE 2:**
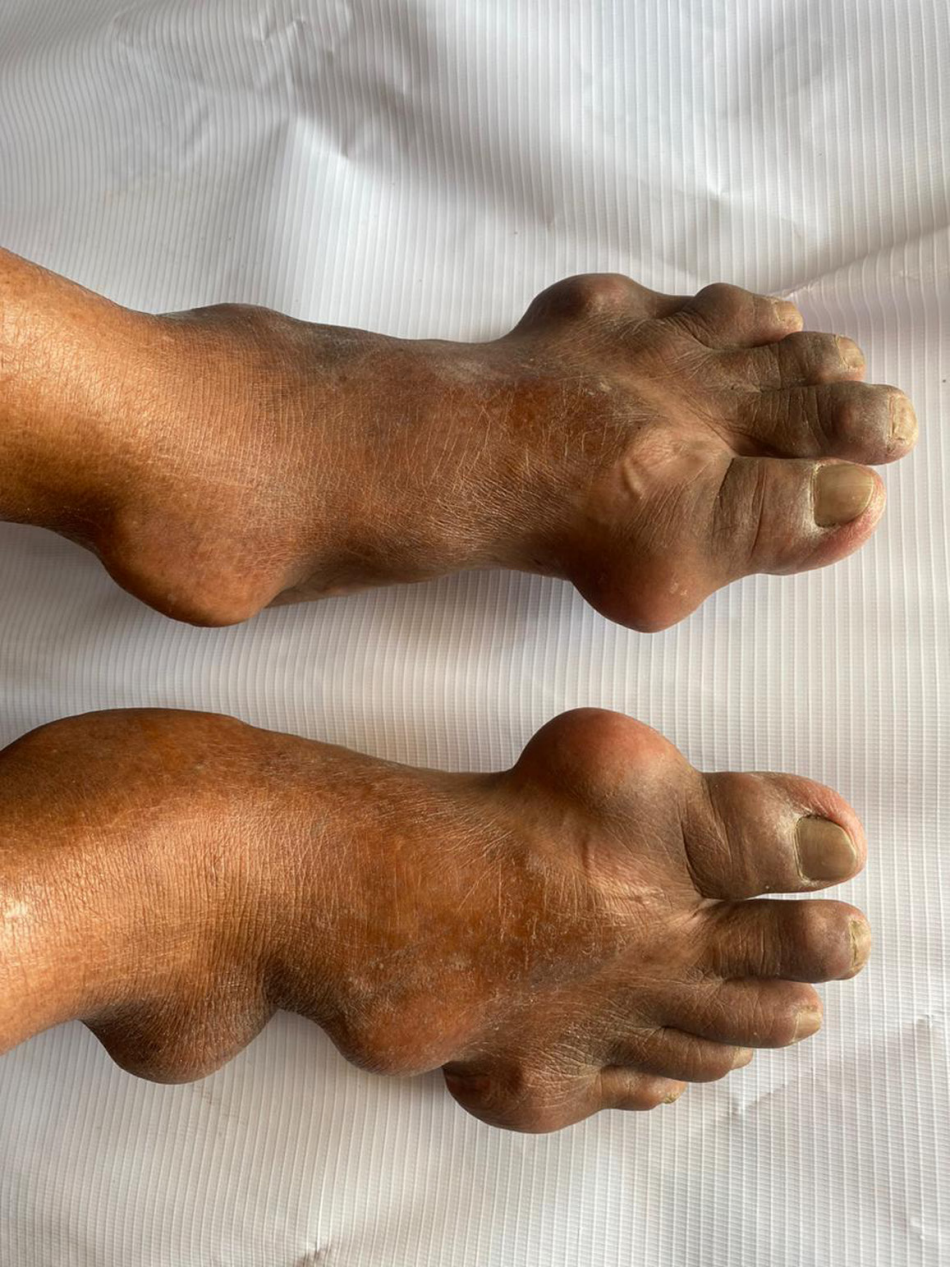
Multiple swellings involving 1st and 5th metatarsophalangeal joints and ankle joints of both feet.

An accumulation of monosodium urate crystals causes gout that most frequently affects the first metatarsophalangeal joint. Risk factors include increased age, alcohol use, osteoarthritis, purine‐rich foods, family or personal history of gout attacks, and medications such as thiazide diuretics for hypertension.^1^ Hyperuricemic patients (≥7.8 mg/dL) can develop polyarticular tophaceous gout from intermittent arthritis if untreated. Acute flares and tophi development can be avoided by lowering blood urate levels with xanthine oxidase inhibitors or uricosuric medications. A target serum uric acid level of <6.0 mg/dL is desirable.^2^ Surgery is only indicated for gout in situations of repeated attacks with deformities, excruciating pain, infection, and joint damage.^3^

## AUTHOR CONTRIBUTIONS

**Durga Neupane:** Conceptualization; data curation; investigation; methodology; resources; validation; visualization; writing – original draft; writing – review and editing. **Nimesh Lageju:** Conceptualization; data curation; methodology; resources; writing – original draft; writing – review and editing. **Lokesh Shekher Jaiswal:** Conceptualization; data curation; methodology; resources; supervision; writing – original draft; writing – review and editing. **Narendra Pandit:** Conceptualization; methodology; resources; validation; writing – original draft; writing – review and editing. **Asim Mahat:** Conceptualization; methodology; resources; writing – original draft; writing – review and editing. **Saransh Ghimire:** Conceptualization; data curation; methodology; resources; supervision; writing – original draft; writing – review and editing. **Upendra Pokhrel:** Conceptualization; methodology; resources; writing – original draft; writing – review and editing. **Sefali Koirala:** Conceptualization; data curation; methodology; writing – original draft; writing – review and editing.

## FUNDING INFORMATION

None.

## CONSENT

Written informed consent was obtained from the patient to publish this report in accordance with the journal's patient consent policy.

## Data Availability

Data pertaining to the manuscript is available upon request to corresponding author.
